# Cognitive and immunological effects of yoga compared to memory training in older women at risk for alzheimer’s disease

**DOI:** 10.1038/s41398-024-02807-0

**Published:** 2024-02-14

**Authors:** Adrienne Grzenda, Prabha Siddarth, Michaela M. Milillo, Yesenia Aguilar-Faustino, Dharma S. Khalsa, Helen Lavretsky

**Affiliations:** 1 Department of Psychiatry & Biobehavioral Sciences, David Geffen School of Medicine at UCLA, and Semel Institute for Neuroscience and Behavior, UCLA, Los Angeles, CA USA; 2 UCLA-Olive View Medical Center, Sylmar, CA USA; 3 Alzheimer’s Research and Prevention Foundation, Tucson, AZ USA

**Keywords:** Long-term memory, Psychiatric disorders

## Abstract

Subjective cognitive decline (SCD) and mild cognitive impairment (MCI) accompanied by cerebrovascular risk factors (CVRFs) are known to increase the risk of developing dementia. Mind-body practices such as yoga and meditation, have been recognized as safe techniques with beneficial effects on cognitive functions in older adults at risk for cognitive decline. We conducted a randomized, controlled trial to assess the efficacy of Kundalini yoga training (KY) compared to memory enhancement training (MET) on mood and cognitive functioning in a group of older women with CVRFs and SCD (clinicaltrials.gov = NCT03503669). The KY intervention consisted of weekly, 60-min in-person classes with a certified instructor for 12 weeks, with a 12-min guided recording for daily homework practice at home. MET involved 12 weekly in-person group classes with 12-min daily homework exercises. Objective and subjective memory performance were the primary outcomes. Peripheral whole blood samples were collected at baseline, 12-weeks, and 24-weeks follow-up for RNA sequencing and cytokine/chemokine assays. A total of 79 patients (KY = 40; MET = 39) were randomized, and 63 completed the 24-week follow-up (KY = 65% completion rate; MET = 95%; χ2(1) = 10.9, *p* < 0.001). At 24-weeks follow-up, KY yielded a significant, large effect size improvement in subjective cognitive impairment measures compared to MET. KYOn a transcriptional level, at 12- and 24-week follow-up, KY uniquely altered aging-associated signatures, including interferon gamma and other psycho-neuro-immune pathways. Levels of chemokine eotaxin-1, an aging marker, increased over time in MET but not KY participants. These results suggest clinical and biological benefits to KY for SCD, linking changes in cognition to the anti-inflammatory effects of yoga.

## Introduction

Alzheimer’s disease (AD) is a widespread, fatal neurodegenerative disease that is largely resistant to current attempts to slow its progression. With increasing focus on prevention, improved characterization of AD’s preclinical phases is urgently needed. Both mild cognitive impairment (MCI) and subjective memory and cognitive decline (SCD) can precede the progression to AD [1]. In a large population-based sample (*N* = 216,838), social isolation, depression, and hypertension were the primary risk factors for SCD [2]. In several studies, SCD predicted beta-amyloid burden by positron emission tomography (PET) scans [3]. Female apolipoprotein E epsilon 4 allele (*APOE-ε4*) carriers with SCD displayed higher odds (OR = 3.34) of a beta-amyloid positive scan than male *APOE-ε4* carriers (OR = 0.37) [4].

Female sex is an intrinsic risk factor for the development of AD. High burden of cardiovascular disease is among the likely contributors to this disparity, given the shared risk factors with AD, including obesity, diabetes, high cholesterol, and psychiatric illness [5]. Females who carry the *APOE-ε4* variant are twice as likely as men to develop AD [6]. *APOE-ε4* also increases the severity of dementia [7]. In young, healthy persons, the *APOE-ε4* allele associates with abnormal neurovascular functioning, blood pressure, and heart rate [8]. Furthermore, women appear more susceptible to stress-induced cardiovascular event [9]. Menopause, with accompanying reductions in circulating estradiol, which possesses neuroprotective properties, may further exacerbate cognitive deficits [10].

Interventions such as yoga and meditation are multi-component mind-body exercises that impact stress response, inflammation, and cellular senescence. By integrating physiological and cognitive processes, mind-body interventions are well-suited for evaluating interventions targeted toward enhancing healthy aging, including cognitive protection. Previous clinical trials from our group have demonstrated the efficacy of Kundalini yoga (KY) in older adults with MCI [11–14]. Compared to memory enhancement training (MET), a mnemonic-based system for boosting information encoding and retrieval, KY participants exhibited short- and long-term benefits in executive functioning, depression, and resilience [11–13].

To date, no such studies have targeted women at high risk for AD (e.g., postmenopausal with SCD and cardiovascular risk factors). Here we investigate the efficacy and underlying neurobiological mechanisms of response to KY compared to MET on memory performance at 12- and 24-weeks in women at high AD risk in a new randomized, controlled trial. Secondary clinical outcomes examined include mood, resilience, and quality of life. We additionally examined genome-wide transcription and cytokine/chemokine changes at 12- and 24-weeks.

## Methods participants

The UCLA Institutional Review Board approved all study procedures (NCT03503669). All participants provided written informed consent. Participants were recruited from the UCLA Neuropsychiatric Hospital inpatient and outpatient services and from community advertising between May 2018 and February 2021. A total of 359 women were assessed for eligibility of which 251 declined to participate or did not meet inclusion criteria. The remaining 108 participants were screened by phone with 29 subsequently excluded for screening cancellation/no show (*n* = 8), failure to meet inclusion criteria (*n* = 10), or drop out prior to randomization (*n* = 11). Of the 79 consented participants, 40 were randomized to the yoga intervention and 39 to the memory training intervention. The CONSORT diagram for the study is shown in Fig. S1.

### Inclusion/exclusion criteria

Eligibility criteria were as follows: 1) age ≥ 50 years with self-reported menopause; 2) self-reported subjective cognitive decline (SCD) from the prior year’s functioning; 3) the presence of one or more cardiovascular risk factors (assessed by the Cerebrovascular Risk Factor Prediction Chart and hematologic testing), which included (a) history of myocardial infarction no less than 6 months prior, (b) prior diagnosis of diabetes, (c) current pharmacological treatment for blood pressure (>140/90), or (d) current pharmacological treatment for hyperlipidemia (LDL > 160); 4) sufficient English proficiency to comprehend the intervention instructions and materials; and 5) sufficient mental capacity to provide informed consent.

SCD is defined as the subjective experience of declining memory function, despite a normal range of memory function using neuropsychological measures. We employed the criteria set by Innes and colleagues [15], which require an individual meeting all of the following criteria; (1) self-reported memory problems within the past 6 months; (2) frequency of memory problems at least once per week; (3) ability to give an example in which memory problems occur in everyday life; (4) belief that one’s memory capacity has declined in comparison to 5–10 years previously; (5) absence of overt cognitive deficits or dementia diagnosis; and (6) concerns/worries regarding memory problems.

Exclusion criteria were as follows: (1) prior history of psychiatric illness, including psychosis, bipolar disorder, drug or alcohol dependence, or a neurological disorder; (2) surgery within the past three months or planned surgery within the next year, as well as unstable medical conditions; (3) disabilities, such as severe visual or hearing impairment; (4) insufficient English proficiency; (5) a diagnosis of dementia by Mini Mental State Examination (MMSE) [16] < = 23 or Clinical Dementia Rating Scale (CDR) [17] > = 0.5; (6) current participation in cognitive training in a therapeutic setting; (7) current treatment with a psychoactive medication; (8) prior experience with Kundalini yoga; or (9) myocardial infarction within the past 6 months. Patients were not excluded for a prior history of major depressive disorder or current antidepressant treatment.

### Interventions

#### Kundalini yoga (KY)

The KY intervention consisted of weekly, 60-min in-person lessons with a certified KY instructor for 12 weeks. Each class of 6–10 participants followed the same structure: (1) tuning in (5 min); (2) warm up (15 min); (3) breathing techniques “Pranayama” (15 min); (4) Kirtan Kriya (12 min); (5) final resting pose “Savasana” (10 min) and closing (3 min). In addition, each participant received a CD containing a 12-minute KK recording with gentle background music and guidance for the exercise sequence. Participants performed this exercise at home every day. They were instructed to chant along with their eyes closed in a seated position, the feet flat on the floor (i.e., relaxed with a straight spine), to visualize a beam of white light entering the center of the top of the head and exiting the middle of the forehead, which is spiritually considered the third eye. While chanting, the thumb of each hand would touch the other fingers sequentially (“mudras”) along with the words “Saa” (thumb touches second finger), “Taa” (middle finger), “Naa” (ring finger), and “Maa” (fifth finger). Saa Taa Naa Maa translates to “Birth, Life, Death, and Rebirth”. The first round is chanted out loud, the next round whispered, the third is thought silently, the fourth is also whispered, and the fifth round is chanted out loud again. This sequence is repeated for 11 min with the last minute of energetic integration and meditation (total 12 min). This technique is thought to engage different senses simultaneously (visualization, vocalization, motor, and sensory stimulation). Furthermore, the chanting and breathing pattern modulate respiratory muscles, lung volume, cardiovascular and autonomic nervous system functions [18].

#### Memory training (MET)

MET involved 12 weekly in-person group classes presented by a qualified memory training instructor. The classes aimed to teach memory strategies, while participants completed weekly homework assignments and handed them in to ascertain participant compliance. MET was developed by researchers at the UCLA Longevity Center. This MET program involves a scripted curriculum for the trainer and a companion workbook for each participant. The detailed standard protocol for MET was derived from evidence-based techniques that use verbal and visual association, as well as practical strategies for memory learning [19, 20]. MET is performed in small group sessions of 6–10 people and includes (1) education about memory; (2) introduction to memory strategies; (3) instruction of the use of specific memory strategies; (4) home practice along with logs to track activity; and (5) the discussion of non-cognitive factors, such as self-confidence, anxiety, and negative expectations. Each weekly session has the same structure; trainers (1) document the number of participants per session, engage patients in alternative treatments, and collect homework completion logs; (2) review the previous homework exercises to reinforce learned techniques; (3) teach new techniques, review, and conduct exercises in the group session; and (4) assign new homework for the following week. Participants were directed to spend approximately 20 min daily on homework and document their activity in their logs. Each group session was devoted to learning and practicing memory techniques, and 15 min were reserved for reviewing the completed homework. Specific techniques taught include the following: verbal associative techniques (such as the use of stories) to remember lists; organizational strategies (categorizing items on a grocery list); visual associative strategies for learning faces and names [21]; learning to implement memory habits to recall where the person placed an item, what recent activities they performed (e.g. locking doors, turning off appliances); and how they can remember future tasks (i.e. appointments).

#### Adherence and side effects

Staff members tracked the attendance of participants for their weekly in-person training classes. Each participant was allowed a maximum of two missed classes. Participants self-reported if they had completed their homework. Completed homework sheets were submitted to staff during class or testing sessions. Additionally, participants were asked not to participate in any other mind-body practices during the trial period, such as Tai Chi, Qi Gong, or yoga. Side effects and adverse events of interventions were monitored using the UKU Side Effect Rating Scale [22].

### Assessments

#### Cognitive domain

A delayed recall domain score was computed from three tests: (1) Hopkins Verbal Learning Test-Revised (delayed recall), (2) Wechsler Memory Scale-IV (Verbal Paired Associated, delayed recall), and (3) Rey-Osterreith Complex Figure delayed recall trial. An executive function domain score was calculated from two tests: (1) Stroop Interference [Golden version] [23], and (2) Trail Making Test B [24]. Trails B was reverse scored such that higher values indicate better performance. Cognitive domain assessments were completed at baseline and 24-week follow-up.

#### Subjective memory

The Memory Functioning Questionnaire (MFQ) assesses subjective memory functioning and consists of 64 items rated on a seven-point scale, and provides four unit-weight factor scores measuring: Factor 1, frequency of forgetting (including ratings of how often forgetting occurs in 28 specific situations and five ratings of general memory performance; 33 items); Factor 2, seriousness of forgetting (memory failure ratings from 18 different situations; 18 items); Factor 3, retrospective functioning (changes in current memory ability relative to five time points earlier in life; 5 items), and Factor 4, mnemonics usage (frequency of mnemonics usage in eight specific situations; 8 items). Higher scores indicate higher levels of perceived memory functioning, i.e., fewer forgetting incidents, less frequent use of mnemonics. Factor structure is stable across age groups and internal consistency is high, with Cronbach’s alpha values for its four factor scores ranging from 0.83 to 0.94 [25]. In the present study, we focused on two of the more commonly used factors, namely the frequency of forgetting (MFQ1), and seriousness of forgetting (MFQ2) which have been shown to more robustly reflect AD pathology than other MFQ components [26]; higher scores indicate better functioning. The MFQ was administered at baseline, 12-week, and 24-week follow-up.

Patients additionally completed the Hamilton Anxiety Rating Scale (HAM-A) [27], Connor-Davidson Resilience Scale (CD-RISC-25) [28], Perceived Stress Scale (PSS) [29], 36-Item Short Form Survey (SF-36), and Beck Depression Inventory (BDI) [30]. These assessments were administered at baseline, 12-week, and 24-week follow-up.

### Outcomes

Primary outcomes of interest were changes in (1) cognitive domain scores (delayed recall, executive functioning) at 24-week follow-up compared to baseline; and (2) subjective memory (MFQ) scores at 12- and 24-week follow-up compared to baseline. Secondary outcomes examined were changes in depression (BDI), anxiety (HAM-A), perceived stress (PSS), resilience (CD-RISC-25), and health-related quality of life (SF-36, all subscales).

### Statistical analysis

Data were entered at the time of collection and analyzed after completion of the trial. All data were inspected for outliers, homogeneity of variance and other assumptions to ensure their appropriateness for parametric statistical tests. Intervention groups were compared using *t*-tests (continuous variables) or chi-squared tests (categorical variables) on all demographic and outcomes measures at baseline. For cognitive domain scores, raw scores were z-transformed for each test according to the study sample’s mean and the *z*-scores were averaged within each domain to produce domain *z*-scores. Continuous outcomes were analyzed using a mixed effects general linear model, as implemented in SAS PROC MIXED, including treatment group, time, and the interaction between time and treatment group. Age, sex, and education (only for cognitive outcomes) were used as covariates. Significance of the interaction between time and intervention group was used to assess whether the groups differed in changes in outcome measures. Post-hoc analyses determined the significance of specific pair-wise group differences and within-group changes. Changes in test scores and statistics as well as effect sizes (Cohen’s d) for group differences are provided. All analyses were conducted using SAS 9.4 (SAS Institute, Cary, North Carolina).

### Cytokine/chemokine assay & analysis

ACD-anticoagulated blood was transported at room temperature and processed within 18 h of blood draw. Whole blood was centrifuged at 2000 rpm for 10 min and plasma immediately stored at −80 °C. Human 38-plex magnetic cytokine/chemokine kits (EMD Millipore, HCYTMAG-60K-PX38, Burlington, MA) were used per manufacturer’s instructions and as previously described [31, 32]. The panel includes IL-1RA, IL-10, IL-1α, IL-1β, IL-6, IFN-α2, TNF/TNF-α, TNF-β/LT-α, sCD40L, IL-12p40, IFN-γ, IL-12/IL-12p70, IL-4, IL-5, IL-13, IL-9, IL-17A, GRO/CXCL1, IL-8/CXCL8, eotaxin-1/CCL11, MDC/CCL22, fractalkine/CX3CL1, IP-10/CXCL10, MCP-1/CCL2, MCP-3/CCL7, MIP-1α/CCL3, MIP-1β/CCL4, IL-2, IL-7, IL-15, GM-CSF, Flt-3L/CD135, G-CSF, IL-3, EGF, FGF-2, TGF-α, and VEGF. Fluorescence was quantified using a Luminex 200™ instrument (Austin, TX). Cytokine/chemokine concentrations were calculated using Milliplex Analyst software version 4.2 (EMD Millipore, Burlington, MA). Luminex assay and analysis were performed by the UCLA Immune Assessment Core. Manufacturer’s recommended quality control procedures were followed to ensure validity. Only those cytokines with no more than 25% of samples were undetectable were included in analyses. Seventeen analytes (EGF, FGF_2, eotaxin-1/CCL11, Flt-3L/CD135, IFN-γ, GRO/CXCL1, IL-10, MCP-3/CCL7, IL-12p40, MDC/CCL22, sCD40L, IL-1RA, IL-8/CXCL8, IP-10/CXCL10, MCP-1/CCL2, MIP-1β/CCL4) were identified in this manner. Cytokine concentration levels were log-transformed before analyses. Significance was set at *p* ≤ 0.05 for all analyses.

### RNA-Sequencing

#### Sample collection & processing

Peripheral whole blood samples were collected at baseline, 12-week, and 24-week follow-up in EDTA-coated tubes. Sample were incubated in red blood cell lysis buffer, washed, pelleted, then stored at –80 °C in RNAprotect Tissue Reagent (Qaigen, Valencia, CA) until processing. Total RNA extraction and cDNA library construction were carried out by the UCLA Technology Center for Genomics & Bioinformatics (TCGB, Los Angeles, CA). 156 samples were sequenced on two Illumina NovaSeq S4 lanes using 150 bp paired-end chemistry (Illumina, San Diego, CA). A total of 2.6 × 10^9^ million reads were generated from 156 RNA samples (mean 33.9 +/− 4.4 (SD) million reads per sample). Prior to read trimming and quality filtering, 83% of all forward and reverse reads had an average quality score ≥ Q30 with a total aligned percentage of 71%.

#### Read trimming, quality filtering, and mapping

The quality of raw paired-end reads was assessed with *fastqc* (v0.11.8) [33] and *multiqc* (v1.13) [34]. Reads were evaluated for insert size, average sequence quality, and percentage GC content. Adapter removal, quality trimming, and filtering (Q ≥ 20, average read quality score ≥25, and read length ≥50 bp), and base corrections were done using *fastp* (v0.23.2) [35]. Quality-processed reads were then re-assessed using *multiqc* to ensure the effectiveness of filtering. Approximately 90% of reads in each sample were ≥Q30 after trimming and. Transcript quantification for the quality-processed reads was estimated using *salmon* (version 1.10.1) in selective alignment mode with the “seqBias” and “gcBias” parameters using a decoy-aware reference transcriptome index (built from Ensembl GRCh38.97) [36]. Read mapping efficacy, by percentage and total reads maps, was assessed using *multiqc* to ensure a minimum of 50% of filtered reads or 10 million total reads mapped to the transcriptome index.

#### Differential gene expression analyses

All subsequent analyses were performed in R (v4.2.3). Transcript per million (TPM) quantifications of transcript abundance were used to estimate gene-level pseudo-read counts with *tximeta* (v1.16.1), which corrects count estimates for library size and read length biases (i.e., within sample normalization) [37]. Hierarchical cluster (single linkage) trees were used to identify outlier samples. A total of 9 samples were discarded. Quantile normalization was used for between-sample normalization using the *qsmooth* approach [38]. Only protein-coding genes were considered for further analysis. Genes with zero counts across all samples were removed, leaving a total of *n* = genes for further analysis. Differential gene expression analysis was performed using *edgeR* (v3.40.2) using treatment and timepoint as a combined covariate (each timepoint for each treatment defining one group level) in a negative binomial generalized linear model evaluated by a quasi-likelihood *F*-test [39]. Genes were considered differentially expressed if log2 fold change >1 and FDR < 0.1.

To assess for differential expression in a threshold-free manner, a stratified Rank-Rank Hypergeometric Overlap (RRHO) test using the RRHO2 package (v1.0) was performed [40]. Differential gene expression lists from the *egdeR* analysis were ranked by their log2 fold change values. The RRHO test calculates a *p*-value for each rank pair, representing the probability of observing the overlap by chance. The rank-rank plot displays the extent and significance of the overlap between the two gene lists. Discordant genes were examined for enrichment using the *enrichR* package (v3.2) using the GTEx_Aging_Signatures_2021 database [41, 42]. Results were ranked based on the adjusted *p*-values (Benjamini-Hochberg method) and the combined score, a measure that considers both the *p*-value and the *z*-score for each enriched term. A term was deemed statistically significant if the adjusted *p*-value was less than 0.05 and the combined score was greater than 1.

#### Weighted gene co-expression network analysis (WGCNA)

WGCNA was performed to identify co-expressed gene modules and investigate their association with phenotypic traits. The analysis was carried out using the WGCNA package (v1.72.1) in R [43]. From normalized counts, to reduce noise, the top 5000 genes by coefficient of variance were selected (CV = (standard deviation / mean) × 100%). To construct the signed co-expression network, a pair-wise Pearson’s correlation matrix was calculated across all samples for each gene pair. The correlation matrix was subsequently transformed into an adjacency matrix using a power adjacency function, with the soft-thresholding power (β) selected based on the criterion of approximate scale-free topology (R^2^ > 0.8). This was achieved by performing the *pickSoftThreshold* function provided by the WGCNA package, which computes the power that best satisfies the scale-free topology criterion while preserving the connectivity of the network. Next, the adjacency matrix was transformed into a topological overlap matrix (TOM) to capture the relative interconnectedness of genes within the network. The TOM-based dissimilarity matrix (1-TOM) was used for average linkage hierarchical clustering, and gene modules were identified using the dynamic tree cut algorithm with the following parameters: minimum module size of 30 genes, deepSplit = 4, and cut height = 0.2. Module eigengenes (MEs) were calculated as the first principal component of each module’s expression data, which represents the overall gene expression profile of the module. To associate the gene modules with phenotypic traits, Pearson’s correlation coefficients were calculated between MEs and the traits of interest. Modules with significant correlations (*p* < 0.1) were considered associated with the given traits.

### RESULTSKY and MET are well-tolerated interventions in postmenopausal women with cardiovascular risk factors and subjective cognitive impairment

The baseline demographic, clinical, and cognitive characteristics of the randomized sample (*N* = 79) are summarized in Table 1 by treatment group, either KY (KY, *N* = 40) or Memory Enhancement Training (MET, *N* = 39). The mean age of all participants at baseline was 66.5 (SD = 9.2) years, mean BMI was 27.2 (SD = 6.0), mean *CVRF* was 10.1 (SD = 4.6), and mean *MMSE* was 28.4 (SD = 1.4). At baseline, treatment groups did not differ significantly in age, race, years of education, BMI, *CVRF*, *HAM-A*, *MFQ*, *CD-RISC*, *PSS*, *SF-36*, and *BDI*. Two participants (2.5% of the sample, 1 KY and 1 MET) met criteria for MCI at baseline (defined as scoring >1 standard deviation below normal on Hopkins Verbal Learning Test-Revised or Rey-Osterreith delayed recall). No significant differences were noted in cognitive domain scores between the two treatment groups. Twenty-six KY (65%) and 37 MET (95%) participants completed the trial and post-treatment assessment at 6 months (χ^2^(1) = 10.9, *p* < 0.001). Pre-intervention dropout rate did not significantly differ between the 2 arms (5 (12.5%) KY and 1 MET (2.6%), χ^2^(1) = 2.8, *p* = 0.1) but differences were noted in discontinuation during intervention (9 KY (25.7%) and 1 MET (2.6%), χ^2^(1) = 10.9, *p* < 0.001). Tolerability and number of side effects also did not differ. Class attendance for the two treatment arms were comparable.

**Table 1:** Baseline demographic, clinical, and cognitive characteristics of trial participants.

Characteristic	KY (*N* = 40) Mean (SD) or *N*(%)	MET (*N* = 39) Mean (SD) or *N*(%)
Demographics		
Age, years	65.45 (9.11)	67.54 (9.30)
Race		
White	27 (68%)	25 (64%)
Black	4 (10%)	6 (15%)
Asian	2 (5%)	5 (13%)
Hispanic	5 (13%)	1 (3%)
Other	2 (5%)	2 (5%)
Right-handed	32 (80%)	33 (85%)
Education, years	16.15 (1.90)	15.72 (1.99)
Clinical outcomes		
BDI	7.28 (4.73)	7.49 (5.06)
BMI	26.44 (5.13)	28.01 (6.75)
CD-RISC-25	76.31 (11.68)	74.74 (13.78)
CVRF	9.72 (4.87)	10.44 (4.37)
HAM-A	4.50 (2.48)	5.21 (3.41)
MMSE	28.46 (1.71)	28.41 (1.09)
MFQ		
1-Frequency of Forgetting	4.46 (1.01)	4.51 (1.16)
2-Seriousness of Forgetting	4.06 (1.33)	4.37 (1.27)
PSS	21.65 (3.92)	21.36 (4.81)
SF-36		
Physical functioning	68.82 (24.75)	64.36 (27.44)
Role limitations (physical)	63.16 (40.58)	70.51 (38.42)
Role limitations (emotional)	67.54 (41.36)	82.91 (28.48)
Energy/vitality	58.68 (20.32)	60.13 (21.47)
Emotional well-being	78.21 (13.78)	76.82 (14.60)
Social functioning	77.96 (20.84)	79.17 (21.90)
Bodily pain	73.62 (20.42)	74.36 (22.10)
General health perception	71.32 (15.71)	66.79 (18.37)
Cognitive Domains		
Delayed recall	0.14 (0.90)	−0.14 (1.07)
Executive functioning	0.08 (0.89)	−0.07 (1.10)

*KY* Kundalini Yoga & Kirtan Kriya + Meditation, *MET* Memory Enhancement Training, *BMI* Body mass index, *CVRF* Cardiovascular risk factors, *HAM-A* Hamilton Anxiety Rating Scale, *MMSE* Mini Mental State Examination, *MFQ* Memory Functioning Questionnaire, *CD-RISC-25* Connor-Davidson Resilience Scale, *PSS* Perceived Stress Scale, *SF-36* 36-Item Short Form Survey, *BDI* Beck Depression Inventory, *CI* Confidence Interval.

### KY participants experienced long-term benefits in subjective memory measures compared to MET participants but reduced delayed recall

Changes in all outcome measures at 12 weeks and 24 weeks from baseline for the two study arms as well as between-group and within-group statistics are presented in Table 2 and estimated effect sizes (Cohen’s d) with associated 95% confidence intervals are presented in Table 3. At 12-weeks and 24-weeks follow-up, both interventions demonstrated improvement in frequency of forgetting (MFQ-Factor 1). Between group differences, however, were not significant (F(1, 76) = 0.2, *p* = 0.7). At 24-weeks, KY participants demonstrated between- and within-groups improvements in seriousness of forgetting/MFQ-Factor 2 (KY mean change (SD) = 0.65 (1.25), t(76) = 2.1, *p* = 0.04; MET mean change (SD) = −0.31 (1.35), t(76) = −0.9, *p* = 0.4; F(1, 76) = 4.9, *p* = 0.03; effect size (95% confidence interval) = −0.73 (−1.26, −0.19)). KY participants demonstrated between- and within-group decline in delayed recall scores at 24-weeks (KY mean change (SD) = −0.31 (0.37 t(76) = −3.8, *p* = 0.0003; MET mean change (SD = 0.02 (0.55), t(76) = 0.5, *p* = 0.6; F(1, 76) = 10.3, *p* = 0.002; effect size (95% confidence interval = 0.69 (0.17, 1.21)). Executive functioning, however, showed no between- or within-groups differences (F(1, 76) = 0.8, *p* = 0.4). Removing the two participants with MCI did not change the direction or significant of any results (data not shown). Significant differences were not observed among secondary outcomes at 12-week or 24-weeks, within or between groups, save for a within-group decrease in SF-36 Role limitations (emotional) subscore for MET only at 24-week follow-up.

**Table 2:** Changes in outcome measures at 12- and 24-week follow-up.

Outcome, 12 W follow-up	KY (*N* = 26) Mean diff. (SD)^a^	Within-Group Statistics	MET (*N* = 37) Mean diff. (SD)^a^	Within-Group Statistics	Between-Group Statistics
BDI	−1.22 (4.71)	t(76) = −0.9, *p* = 0.4	−0.41 (5.48)	t(76) = −0.5, *p* = 0.6	F(1, 76) = 0.1, *p* = 0.8
CD-RISC	1.87 (10.36)	t(76) = 0.2, *p* = 0.8	0.37 (8.39)	t(76) = 0.2, *p* = 0.9	F(1, 76) = 0.0, *p* = 0.9
CVRF	0.25 (2.21)	t(76) = 0.5, *p* = 0.6	−0.34 (2.11)	t(76) = −1.0, *p* = 0.3	F(1, 76) = 1.1, *p* = 0.3
HAM-A	−2.04 (3.41)	t(76) = −2.0, *p* = 0.05	−1.03 (3.83)	t(76) = −1.9, *p* = 0.06	F(1, 76) = 0.1, *p* = 0.8
MFQ					
1-Frequency of Forgetting	0.63 (0.87)	t(76) = 2.9, *p* = 0.01	0.25 (0.70)	t(76) = 2.1, *p* = 0.04	F(1, 76) = 0.7, *p* = 0.4
2-Seriousness of Forgetting	0.23 (1.33)	t(76) = −0.5, *p* = 0.6	−0.15 (1.33)	t(76) = −0.3, *p* = 0.8	F(1, 76) = 0.3, *p* = 0.6
PSS	−0.14 (4.22)	t(76) = −0.2, *p* = 0.8	0.34 (6.20)	t(76) = 0.3, *p* = 0.8	F(1, 76) = 0.0, *p* = 1.0
SF-36					
Physical functioning	1.82 (23.28)	t(76) = 0.2, *p* = 0.8	−1.57 (35.45)	t(76) = −0.1, *p* = 0.9	F(1, 76) = 0.1, *p* = 0.8
Role limitations (physical)	−4.55 (28.49)	t(76) = −0.6, *p* = 0.6	−6.43 (45.51)	t(76) = −0.6, *p* = 0.6	F(1, 76) = 0.0, *p* = 0.9
Role limitations (emotional)	0 (41.15)	t(76) = 0.2, *p* = 0.8	−11.43 (37.87)	t(76) = −1.9, *p* = 0.08	F(1, 76) = 1.0, *p* = 0.3
Energy/vitality	−0.68 (18.92)	t(76) = −0.5, *p* = 0.6	1.29 (14.82)	t(76) = 0.6, *p* = 0.5	F(1, 76) = 0.6, *p* = 0.4
Emotional well-being	−3.09 (17.89)	t(76) = −1.3, *p* = 0.2	0.69 (14.98)	t(76) = 0.2, *p* = 0.8	F(1, 76) = 1.3, *p* = 0.3
Social functioning	−2.84 (24.38)	t(76) = −0.7, *p* = 0.5	1.43 (27.75)	t(76) = 0.5, *p* = 0.6	F(1, 76) = 0.6, *p* = 0.4
Bodily pain	−3.86 (18.62)	t(76) = −1.2, *p* = 0.2	−1.64 (16.55)	t(76) = −0.1, *p* = 0.9	F(1, 76) = 0.8, *p* = 0.4
General health perception	−1.59 (18.35)	t(76) = −0.8, *p* = 0.4	0.71 (12.49)	t(76) = 0.4, *p* = 0.7	F(1, 76) = 0.8, *p* = 0.4

Outcome, 24 W follow-up	KY (*N* = 26) Mean diff. (SD)^b^	Within-Group Statistics	MET (*N* = 37) Mean diff. (SD)^b^	Within-Group Statistics	Between-Group Statistics
BDI	−1.69 (5.57)	t(76) = −1.3, *p* = 0.2	−0.79 (4.79)	t(76) = −1.2, *p* = 0.2	F(1, 76) = 0.0, *p* = 0.9
CD-RISC-25	−1.00 (13.52)	t(76) = −0.9, *p* = 0.4	−0.39 (11.53)	t(76) = −0.2, *p* = 0.8	F(1, 76) = 0.3, *p* = 0.6
HAM-A	−0.15 (5.15)	t(76) = −0.4, *p* = 0.7	−1.14 (3.7)	t(76) = −1.8, *p* = 0.07	F(1, 76) = 2.4, *p* = 0.1
MFQ					
1-Frequency of Forgetting	0.54 (1.03)	t(76) = 2.7, *p* = 0.01	0.3 (0.75)	t(76) = 2.3, *p* = 0.02	F(1, 76) = 0.2, *p* = 0.7
2-Seriousness of Forgetting	0.65 (1.25)	t(76) = 2.1, *p* = 0.04	−0.31 (1.35)	t(76) = −0.9, *p* = 0.4	F(1, 76) = 4.9, *p* = 0.03
PSS	−0.33 (4.47)	t(76) = −0.2, *p* = 0.8	−0.03 (6.31)	t(76) = 0.0, *p* = 1.0	F(1, 76) = 0.0, *p* = 0.8
SF-36					
Physical functioning	2.71 (20.48)	t(76) = 0.3, *p* = 0.8	0.61 (26.48)	t(76) = 0.5, *p* = 0.6	F(1, 76) = 0.0, *p* = 0.9
Role limitations (physical)	3.12 (39.91)	t(76) = 0.3, *p* = 0.7	−15.91 (40.42)	t(76) = −1.9, *p* = 0.07	F(1, 76) = 2.2, *p* = 0.1
Role limitations (emotional)	2.78 (46.02)	t(76) = 0.2, *p* = 0.9	−18.18 (44.17)	t(76) = −2.5, *p* = 0.01	F(1, 76) = 3.1, *p* = 0.1
Energy/vitality	0.83 (18.8)	t(76) = 0.0, *p* = 1.0	−2.42 (18.96)	t(76) = −0.6, *p* = 0.6	F(1, 76) = 0.1, *p* = 0.7
Emotional well-being	−3.33 (19.01)	t(76) = −1.2, *p* = 0.2	0.73 (14.02)	t(76) = 0.2, *p* = 0.9	F(1, 76) = 1.0, *p* = 0.3
Social functioning	−9.9 (36.3)	t(76) = −1.8, *p* = 0.1	0 (24.61)	t(76) = 0.2, *p* = 0.9	F(1, 76) = 2.0, *p* = 0.2
Bodily pain	−7.5 (25.31)	t(76) = −0.5, *p* = 0.6	−4.92 (17.36)	t(76) = −1.2, *p* = 0.2	F(1, 76) = 0.2, *p* = 0.7
General health perception	−1.46 (16.18)	t(76) = −0.2, *p* = 0.8	1.06 (13.33)	t(76) = 0.5, *p* = 0.6	F(1, 76) = 0.8, *p* = 0.4
*Cognitive Domains*					
Delayed recall	−0.31 (0.37)	t(76) = −3.8, *p* = 0.0003	0.02 (0.55)	t(76) = 0.5, *p* = 0.6	F(1, 76) = 10.3, *p* = 0.002
Executive functioning	−0.04 (0.42)	t(76) = −0.9, *p* = 0.4	−0.03 (0.7)	t(76) = −0.4, *p* = 0.7	F(1, 76) = 0.8, *p* = 0.4

^a^Mean difference = 12-week follow-up score – Baseline score.

^b^Mean difference = 24-week follow-up score – Baseline score.

*KY* Kundalini Yoga & Kirtan Kriya + Meditation, *MET* Memory Enhancement Training, *BMI* Body mass index, *CVRF* Cardiovascular risk factors, *HAM-A* Hamilton Anxiety Rating Scale, *MMSE* Mini Mental State Examination, *MFQ* Memory Functioning Questionnaire, *CD-RISC-25* Connor-Davidson Resilience Scale, *PSS* Perceived Stress Scale, *SF-36* 36-Item Short Form Survey, *BDI* Beck Depression Inventory.

**Table 3:** Effect size estimates for between group changes in outcome measures at 12- and 24-week follow-up.

Measure	12-week follow-up Effect size (95% CI^)a,b^	24-week follow-up Effect size (95% CI)^a,b^
Clinical		
BDI	0.16 (−0.37, 0.68)	0.17 (−0.34, 0.69)
CD-RISC-25	−0.16 (−0.69, 0.36)	0.05 (−0.47, 0.57)
CVRF	−0.28 (−0.80, 0.25)	—
HAM-A	0.28 (−0.24, 0.79)	−0.23 (−0.73, 0.28)
MFQ		
1-Frequency of Forgetting	−0.49 (−1.03, 0.05)	−0.28 (−0.80, 0.25)
2-Seriousness of Forgetting	−0.28 (−0.82, 0.25)	−0.73 (−1.26, −0.19)
PSS	0.09 (−0.45, 0.62)	0.05 (−0.47, 0.57)
SF-36		
Physical functioning	−0.11 (−0.64, 0.43)	−0.09 (−0.61, 0.44)
Role limitations (physical)	−0.05 (−0.58, 0.49)	−0.47 (−1.00, 0.06)
Role limitations (emotional)	−0.29 (−0.83, 0.25)	−0.47 (−1.00, 0.07)
Energy/vitality	0.12 (−0.41, 0.65)	−0.17 (−0.70, 0.36)
Emotional well-being	0.23 (−0.30, 0.77)	0.25 (−0.28, 0.78)
Social functioning	0.16 (−0.37, 0.69)	0.33 (−0.20, 0.86)
Bodily pain	0.13 (−0.41, 0.66)	0.12 (−0.40, 0.65)
General health perception	0.15 (−0.38, 0.69)	0.17 (−0.36, 0.70)
Cognitive Domains		
Delayed recall	—	0.69 (0.17, 1.21)
Executive functioning	—	0.01 (−0.49, 0.52)

^a^Effect sizes are Cohen’s d estimates. For BDI, CVRF, HAM-A, and PSS, where a higher score represents worse symptoms, a positive value indicates a better treatment effect of KY versus MET. For CD-RISC, SF-36, MFQ, and the cognitive domains, where a higher score represents better resilience/performance, a negative value indicates a better treatment effect of KY versus MET.

^b^Cohen’s relative sizes: d = 0–0.19 (trivial); d = 0.2–0.49 (small); d = 0.5–0.79 (medium); d = 0.8+ (large).

*KY* Kundalini Yoga & Kirtan Kriya + Meditation, *MET* Memory Enhancement Training, *BMI* Body mass index, *CVRF* Cardiovascular risk factors, *HAM-A* Hamilton Anxiety Rating Scale, *MMSE* Mini Mental State Examination, *MFQ* Memory Functioning Questionnaire, *CD-RISC-25* Connor-Davidson Resilience Scale, *PSS* Perceived Stress Scale, *SF-36* 36-Item Short Form Survey, *BDI* Beck Depression Inventory, *CI* Confidence Interval.

### Subjective cognitive decline measures associate with underlying gene expression signatures at baseline

**Fig. 1:**
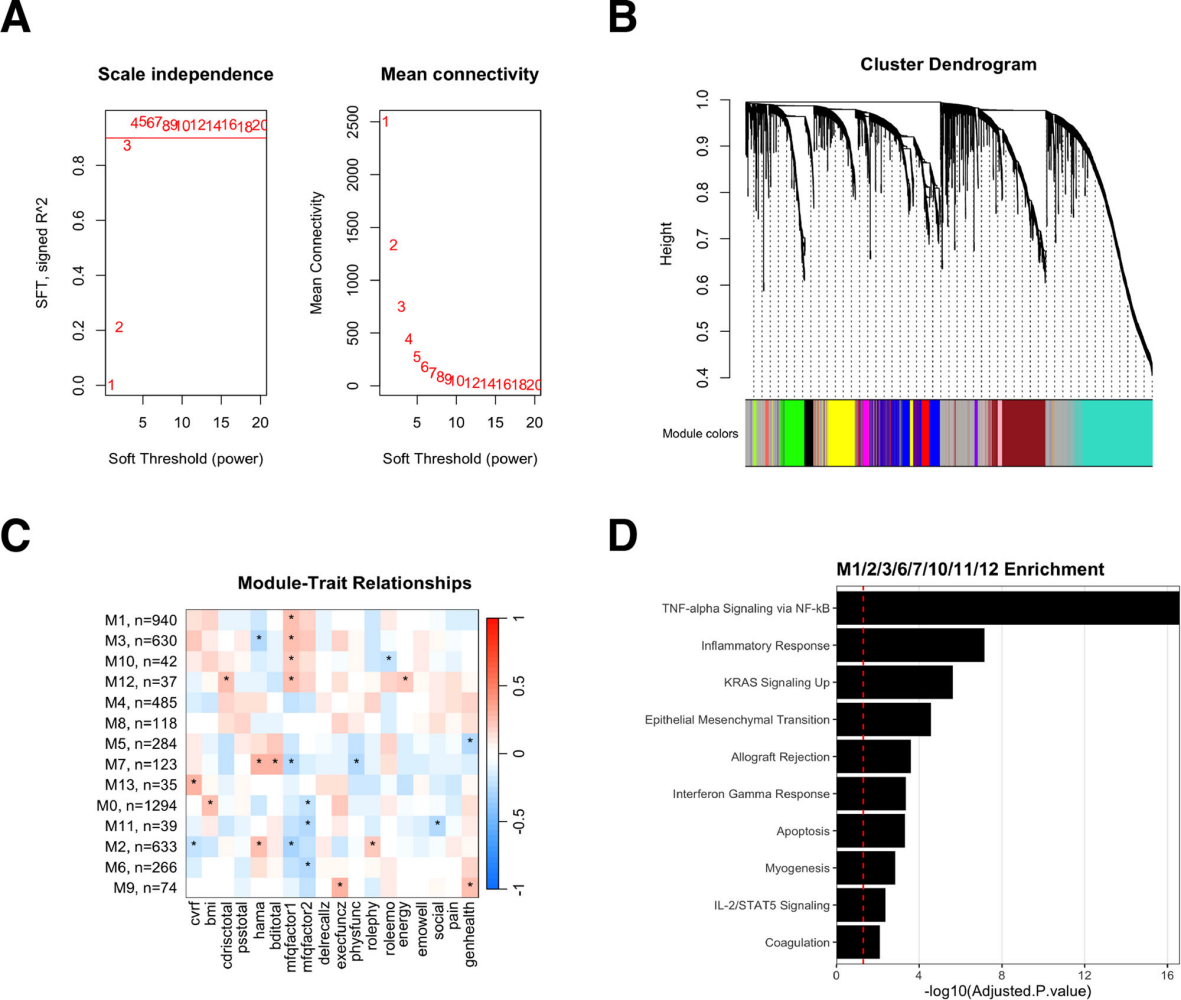
Weighted Gene Co-expression Network Analysis (WGCNA) to characterize module-trait relationships to baseline clinical outcome measures. **A** Scale-free topology criterion plots show the scale independence (left) and mean connectivity (right) of the signed network as functions of the soft-thresholding power. The chosen soft-thresholding power (β) is indicated by a horizontal dashed line, which ensures scale independence above 0.8 and minimum mean connectivity. **B** Cluster dendrogram of the genes, with branches color-coded to represent distinct modules identified by the dynamic tree cut method. The horizontal dashed line represents the threshold for merging modules with a dissimilarity of less than 0.2. **C** Heatmap of module-trait relationships, with each cell displaying the correlation coefficient (Pearson’s r) between the module eigengene and the specific trait (columns). The color gradient ranges from green (negative correlation) to red (positive correlation), with a color key and correlation values provided in each cell. Statistically significant relationships (*p* < 0.1) are marked with an asterisk (*). **D** Over-representation analysis for Module 3, with the x-axis showing the -log10(adjusted *p*-value) and the *y*-axis displaying the top enriched pathways. The horizontal dashed line indicates the significance threshold at adjusted *p*-value < 0.05.

Weighted gene co-expression network analysis (WGCNA) was conducted on baseline gene expression from all participants to examine if distinct gene expression signatures underscore outcome measures at baseline. Prior to the analysis, the suitability of the data for WGCNA was assessed by examining the scale-free topology index (R²) and found it to be greater than 0.8, indicating a scale-free network structure (Fig. 1A, B). The subjective memory outcomes, MFQ1 (frequency of forgetting) and MFQ2 (seriousness of forgetting) demonstrated significant association with 8 modules with the overall pattern of correlation to modules being similar for both measures (Fig. 1C). The genes from these modules were combined and enrichment analysis performed using the MSigDB Hallmark Pathways dataset. The analysis revealed that the modules associated with the subjective memory measures were enriched for pathways related to TNF-alpha signaling, inflammatory response, KRAS signaling, interferon gamma response, apoptosis, and IL-2/STAT5 signaling (Fig. 1D).

### KY participants demonstrate reversal of aging-associated gene expression signatures

**Fig. 2:**
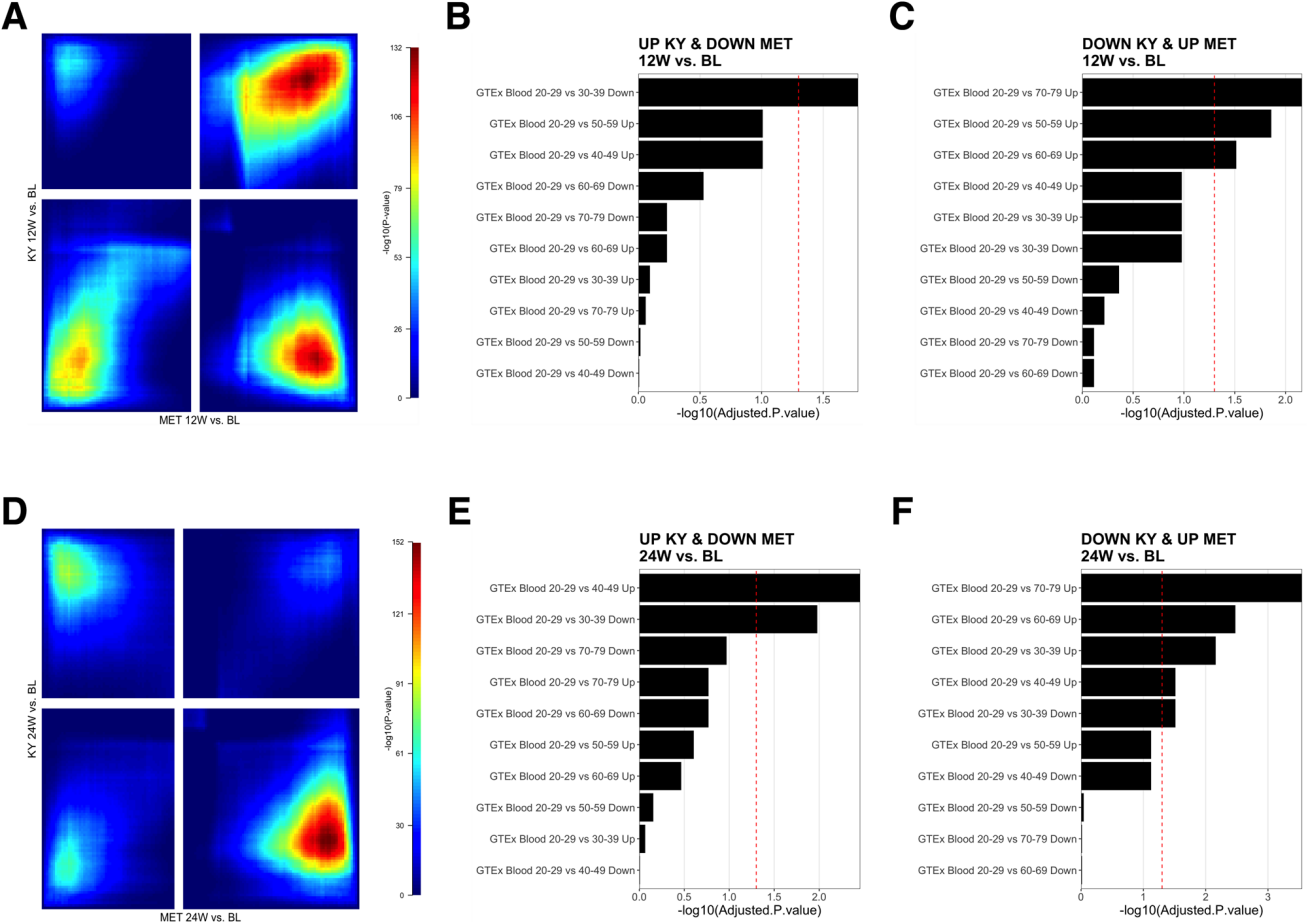
Identification of genes with discordant expression following KY and MET at 12- and 24-weeks enriched for aging signatures. **A** Stratified Rank-Rank Hypergeometric Overlap (RRHO) result map illustrating the statistical significance of the overlap between KY 12-week follow-up versus baseline and MET 12-week follow-up versus baseline. The color gradient represents the -log10 (*p*-value) of the hypergeometric test, with warmer colors indicating higher significance. The *x*- and *y*-axes correspond to the ranked gene lists. Signal in the upper left quadrant and low right quadrant indicates discordant expression (e.g., upregulated in one-dataset and down-regulated in the other). Signal in the upper right quadrant and lower left quadrant (greyed out) represent areas of expression overlap (e.g., upregulated or downregulated in both datasets). **B**, **C** Over-representation analyses of Genotype-Tissue Expression (GTEx) blood aging signatures using discordant genes, where expression is upregulated in KY and downregulated in MET at 12-week follow-up, or vice versa. The x-axis denotes the -log10(adjusted *p*-value) of the analysis result. The red dashed line represents the threshold for statistical significance (adjusted *p*-value < 0.05). **D** Stratified Rank-Rank Hypergeometric Overlap (RRHO) result map illustrating the statistical significance of the overlap between KY 24-week follow-up versus baseline and MET 24-week follow-up versus baseline. **E**, **F** Over-representation analysis of Genotype-Tissue Expression (GTEx) blood aging signatures using discordant genes, where expression is upregulated in KY and downregulated in MET at 24-week follow-up, or vice versa.

To examine differences in gene expression induced by the two interventions, rank-rank hypergeometric (RRHO) analysis was performed to identify discordant gene expression patterns in response to KY and MET treatments at 12- and 24-weeks following intervention initiation compared to baseline (Fig. 2A, D). The RRHO test is a robust, threshold-free method for comparing ranked gene lists, as it considers the rank order of genes while estimating the significance of the overlap between the lists, accounting for the global patterns of gene expression changes. A total of 1123 genes were expressed in a discordant fashion between treatments at 12-week follow-up (307 repressed following KY but overexpressed with MET; 816 overexpressed following KY intervention but repressed with MET), and 1338 genes were discordant at 24 weeks (500 repressed following KY but overexpressed following MET; 838 overexpressed following KY intervention but repressed with MET). Enrichment analysis was performed using the Genotype-Tissue Expression (GTEx) aging signatures database, which characterizes patterns of gene expression at progressively increasing ages compared to a baseline of 20–29 years [9, 44].

At 12-weeks and 24-weeks post-intervention (Supplemental Table S1), discordant genes demonstrated significant enrichment (FDR < 0.05) of aging signatures expressed in the opposing direction of that observed during aging (e.g., genes upregulated in older age were repressed following KY intervention, or vice versa, Fig. 2 B–C, E, F). At 12- and 24-weeks post-treatment, discordant genes were enriched for the 70–79 aging signature in a pattern opposing that observed following the KY intervention (12 W Combined Score = 25.2, adjusted *p*-value = 0.007; 24 W: Combined Score=42.4, adjusted *p*-value < 0.0001). Thus, at 12-week follow-up, KY participants demonstrated significant downregulation of *SEC14L3*, *CPB2*, *IFNG*, *ANKRD33*, *SAA4*, *CCL4*, *CCL3*, *APOA1*, *KIR3DL1, AKR1C4*, *BAAT*, and *SLC38A3*, which are upregulated in the 70–79 aging signature and significantly upregulated in MET participants compared to baseline. At 24-week follow-up, KY participants demonstrated significant downregulation of *PAQR9*, *IL22*, *OR52H1*, *CCL4L2*, *ANKRD33*, *CCL3L3*, *SAA4*, *APOA1*, *BAAT*, *F2*, *BARX1*, *CXCL12*, *C9*, *CFHR3*, *CCL4*, *CCL3*, *HAO1*, *CTSE*, *SLC38A3*, *ACTRT3*, which are upregulated in the 70–79 aging signature and significantly upregulated in MET participants compared to baseline. MET discordant genes were not significantly enriched for any aging signature at 12-week follow-up. At 24-week follow-up, MET participants displayed downregulation of the 40–49 upregulated aging signature (Combined Score = 18.6, adjusted *p*-value = 0.003) and upregulation of the 30–39 downregulated aging signature (Combined Score = 12.8, adjusted *p*-value = 0.004). Differentially expressed genes using a conventional threshold-based approach were also reviewed (Supplementary Fig. S2).

### MET, but not KY participants, demonstrated increased levels of aging-associated chemokine exotoxin-1

No differences in cytokine/chemokine concentrations in peripheral blood were detected between interventions at baseline (Table 4). At 12- and 24-week follow-up, MET participants displayed a significant increase in exotoxin-1 concentrations (MET 12 W: t(67) = −2.12, *p* = 0.04; MET 24 W: t(67) = −2.12, *p* = 0.04). Levels in KY participants were unchanged. The between group difference was significant (F(2,67) = 3.94, *p* = 0.02). Both MET and KY participants demonstrated FGF increases at 12-week follow-up (KY: t(67) = −2.5, *p* = 0.01; MET: t(67) = −2.4, *p* = 0.02), but the between group difference was not significant. No baseline cytokine concentrations were predictive of changes in cognitive domain at 24-week follow-up or MFQ scores 12- or 24-week follow-up.

**Table 4:** Changes in cytokine concentrations at 12- and 24-week follow-up.

Cytokines, 12-week follow-up	KY (*N* = 18) Mean diff. (SE)^a^	Within-Group Statistics	MET (*N* = 27)Mean diff. (SE)^a^	Within-Group Statistics	Between-Group Statistics
Eotaxin-1	0.07 (0.07)	t(65) = 1.06, *p* = 0.32	0.12 (0.05)	t(65) = 2.15, *p* = 0.04	F(1,65) = 0.28, *p* = 0.6
FGF	0.45 (0.18)	t(65) = 2.51, *p* = 0.01	0.38 (0.17)	t(65) = 2.30, *p* = 0.02	F(1,65) = 0.10, *p* = 0.8

Cytokines, 24-week follow-up	KY (*N* = 26) Mean diff. (SE)^b^	Within-Group Statistics	MET (*N* = 32) Mean diff. (SE)^b^	Within-Group Statistics	Between-Group Statistics
Eotaxin-1	−0.09 (0.07)	t(67) = −1.31, *p* = 0.19	0.16 (0.06)	t(67) = 2.70, *p* = 0.01	F(1,67) = 7.65, *p* = 0.01
FGF	0.25 (0.21)	t(67) = 1.17, *p* = 0.3	0.13 (0.19)	t(67) = 0.69, *p* = 0.5	F(1,67) = 0.18, *p* = 0.7

^a^Mean difference = 12-week follow-up score – Baseline score; log-transformed values

*KY* Kundalini Yoga & Kirtan Kriya + Meditation, *MET* Memory Enhancement Training.

## Discussion

In a clinical trial of KY compared to MET in postmenopausal women at elevated risk for developing AD, both interventions resulted in improvements in frequency of forgetting at 12 and 24-week follow-up evaluation compared to baseline, although no between groups differences. At 24-weeks, only KY participants also demonstrated significant within and between group improvements in seriousness of forgetting. Neither intervention resulted in changes in anxiety, depression, perceived stress, or resilience, most likely because participants were relatively healthy and not depressed or anxious. At 24 weeks, delayed recall significantly declined in KY but not MET participants, but neither group experienced decline in executive functioning.

A small subset of patients from the current study were examined by MRI at baseline and 12-weeks. The MET but not KY participants showed grey matter volume reductions in numerous regions at 12 weeks, while only KY participants displayed suggestive increases in hippocampal volume immediately after completion of the intervention [45]. Additionally, connectivity analysis found that the left anterior hippocampal subregion assigned to the default mode network in the Cole-anticevic atlas showed greater increases in connectivity with largely ventral visual stream regions with KY than with MET at 12 weeks (*p* < 0.001) [46]. Altered directed functional connectivity of the hippocampus has been observed in MCI and Alzheimer’s disease [47, 48]. Aging compromises the functional connectivity between the default-mode network regions and other brain areas, including the ventral visual stream regions, which can negatively impact memory and visual perception (object and face recognition) [49, 50]. Interventions that help preserve or bolster this connectivity may be crucial to maintaining cognitive function in the elderly.

In our previous trial comparing KY to MET in older men and women with MCI, both interventions showed improvements in memory. However, KY also resulted in improvements in mood and executive function [11]. MET can improve subjective memory function, although the magnitude of improvement is typically less than that observed by objective measures, a finding replicated in our trials [44]. KY participants uniquely experienced a decline in delayed recall at 24 weeks but not executive functioning. Delayed recall (episodic memory) relies heavily on the medial temporal lobe, including the hippocampus, which is crucial for long-term memory consolidation and retrieval. Executive functions (e.g., working memory, cognitive flexibility, inhibitory control) are primarily associated with the prefrontal cortex. Episodic memory tends to be disproportionality impacted by age-related decline. Therefore, interventions targeting memory in older adults may face greater challenges in stabilizing or improving delayed recall performance, while executive functioning may be more likely to remain stable regardless of intervention.

Alternatively, KY may improve subjective memory by enhancing working memory capacity, while MET may be more effective at strengthening the consolidation and retrieval of long-term memories. Executive function is anticipated to stabilize or slightly improve with either intervention. Furthermore, KY primarily targets memory via improved physical and mental well-being, whereas MET involves cognitive exercises designed to improve function on specific memory tasks. A criticism of MET, in fact, is that gains tend to be limited to the tasks encountered during training and fail to generalize to daily living [51]. The influence of expectation (placebo effect) also cannot be discounted. Finally, KY associates strongly with improvement in anxiety, depression, stress, and well-being, which may potentiate the effects of the intervention on memory. However, such improvements were not observed in the current study, most likely due to the lack of significant distress among the participants.

At baseline, subjective memory measures associated with genes related to psycho-neuro-inflammatory pathways, including IL-10, tumor-necrosis factor-alpha (TNF-alpha), and interferon-gamma (INF-gamma), cytokines previously implicated in neuroplasticity, mood modulation, and cognition in rodent and clinical data [52]. Furthermore, at 12- and 24-weeks post-intervention, KY but not MET participants demonstrated reversal of aging signatures comprised of IL-10 and INF-gamma signaling-related cytokines, including discordant expression of INF-gamma. Interferon-gamma triggers the production of nitric oxide synthase and reactive nitrogen intermediates as well as TNF-alpha from microglia, leading to microvessel inflammation and neuronal dam age [53]. Subjective memory complaints associate with increased levels of INF-gamma [54]. In mouse models AD, chronic intrahippocampal expression of IFN-gamma leads to an increase in microglial activation and associated with the severity of amyloid-related pathology. However, the mice also demonstrate reduced phosphor-tau pathology and evidence of increased neurogenesis [55]. These results support a dual-role for INF-gamma in maintaining neuronal hemostasis and early cognitive decline. This would additionally explain often contradictory results in investigations of the association between INF-gamma and its downstream targets to cognitive decline.

Peripheral protein levels of eotaxin-1 significantly increased over the course of the study in MET group, but not the KY group. Eotaxin-1 (CCL11) is a chemokine that has been implicated in the selective recruitment of eosinophils into inflammatory sites during allergic reactions. Recent studies have shown that eotaxin-1 can pass through the blood-brain barrier (BBB) and has been identified as a crucial mediator of decreased neurogenesis and cognitive impairment mice [56]. In humans, age-related increases in eotaxin-1 are associated with cognitive impairments in episodic and semantic memory [57]. Given its ability to cross the BBB, peripheral eotaxin-1 may induce neuronal cytotoxicity effects in the central nervous system. Further work is needed to determine if eotaxin-1 is a prognostic biomarker or target for therapeutic interventions.

We must acknowledge the limitations of our study, which include: (1) Modest sample size and high homogeneity among participants, which may have limited the ability to detect smaller effects between interventions. Furthermore, results may not generalize to the broader population of postmenopausal women. (2) Short duration of the intervention and follow-up, which may have been insufficient to precipitate or detect long-term cognitive benefits, respectively. (3) Lack of a usual care arm, preventing estimation of age-related cognitive decline within the time of the follow up or the role of practices effects. (4) Participant adherence to the home practice components may vary from self-report and impact an intervention’s “dose.” (5) Unaccounted differences between groups, such as differing proportions of the APOε4 carriers, may explain the observed group differences in cognitive decline. (6) GLMs produce unbiased estimates provided observations are missing at random; potential effects from data missing not at random cannot be ruled out.

## Conclusion

Cumulatively, our findings suggest that KY can have a positive impact on subjective cognitive decline in older adults at increased risk for cognitive decline. The long-term effects and efficacy of KY in preventing or delaying AD remain to be established. KY appears to uniquely modulate psycho-neuro-inflammatory and aging pathways compared to MET. Additional work is required to determine the precise relationship of these mediators to early cognitive decline.

### Supplementary information

Supplemental Material

## Data Availability

Available upon request to the corresponding author.

## Code availability

Available upon request to the corresponding author.
