# Supplementary material for: Cognitive and immunological effects of yoga compared to memory training in older women at risk for alzheimer’s disease
Source: Transl Psychiatry. 2024 Feb 14;14:96. doi: 10.1038/s41398-024-02807-0 (PMC10867110; doi:10.1038/s41398-024-02807-0)
Supplement: Supplementary file 1 — Supplemental Material [file 41398_2024_2807_MOESM1_ESM.docx]

**Supplemental Figure S1. CONSORT diagram.** KY = Kundalini yoga. MET = Memory Enhancement Training.

**
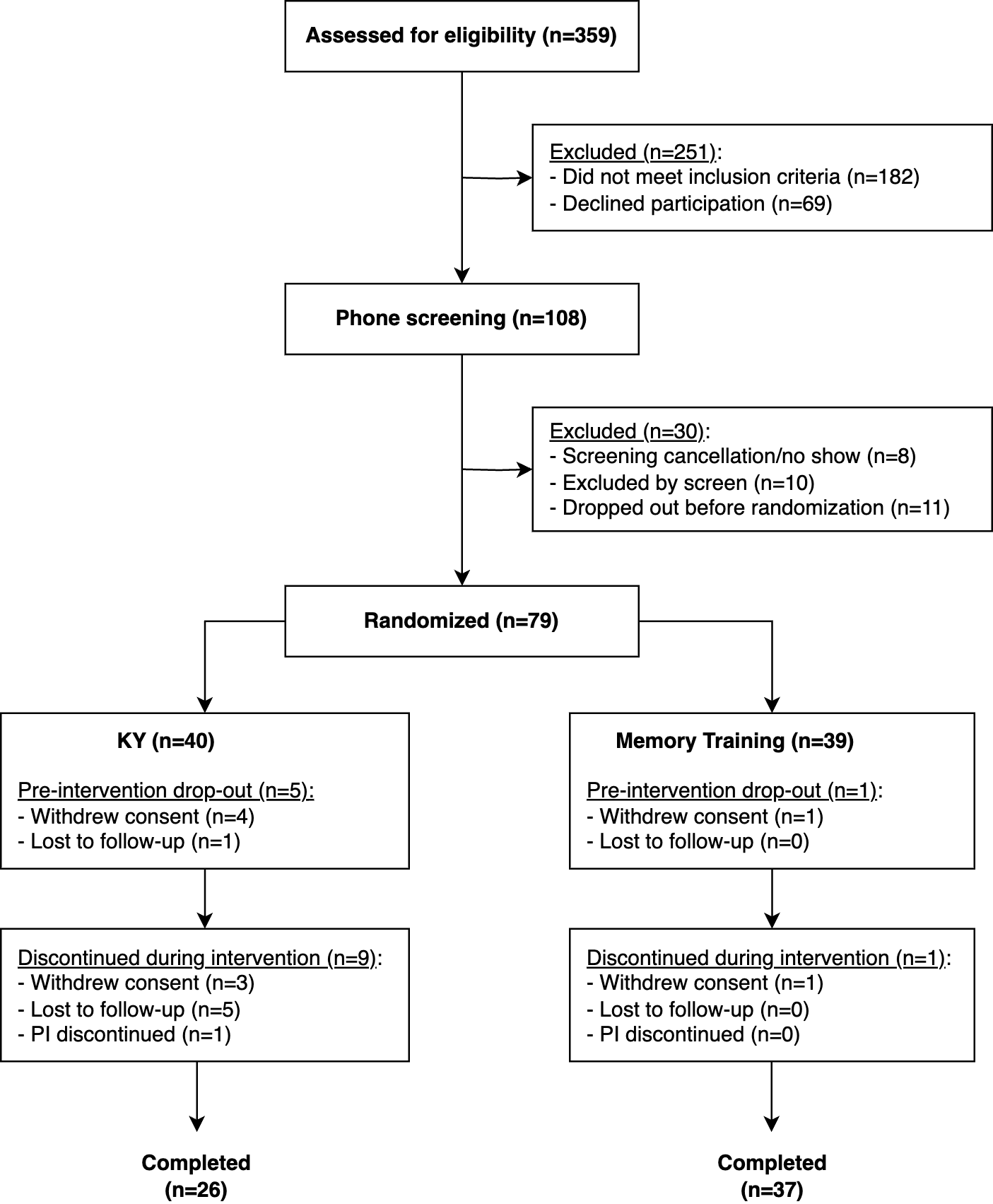
**

**Supplemental Figure 2. Differentially expressed genes (log2 fold change > 1 and FDR <0.1).** (A) MET 12W vs. BL. (B) MET 24W vs. BL. (C) KY 12W vs. BL. (D) KY 24W vs. BL. (E) Upset plot of A-D intersections. KY = Kundalini yoga. MET = Memory Enhancement Training.


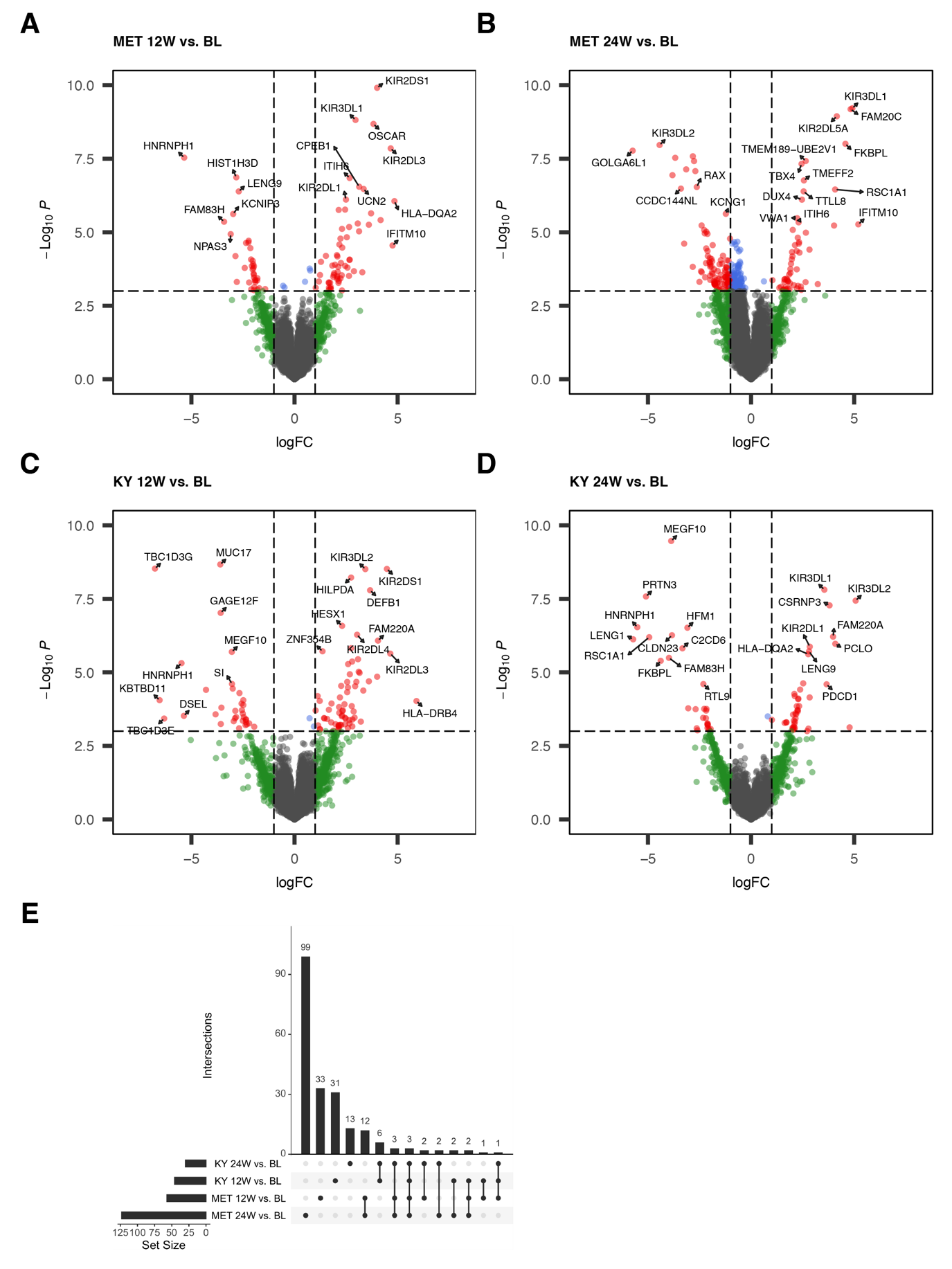
**Supplemental Table 1. GTEx aging signature enrichment by discordant genes.** KY = Kundalini yoga. MET = Memory Enhancement Training.

| **Signature** | **Adj. P-value** | **Score** | **Genes** | **Discordance** |
| --- | --- | --- | --- | --- |
| GTEx Blood 20-29 vs 70-79 Up | 0.0069 | 25.20 | SEC14L3, CPB2, IFNG, ANKRD33, SAA4, CCL4, CCL3, APOA1, KIR3DL1, AKR1C4, BAAT, SLC38A3 | KY Down MET Up 12W |
| GTEx Blood 20-29 vs 50-59 Up | 0.0138 | 19.21 | NLGN4Y, ADAMTS14, ACTN3, IFNG, INSRR, SAA4, CCL4, CCL3, KIR3DL1, AKR1C4, BAAT | KY Down MET Up 12W |
| GTEx Blood 20-29 vs 60-69 Up | 0.0305 | 14.23 | CPB2, IFNG, ANKRD33, KIR2DS4, SAA4, CCL4, CCL3, KIR3DL1, AKR1C4, BAAT | KY Down MET Up 12W |
| GTEx Blood 20-29 vs 30-39 Up | 0.1048 | 6.94 | IFNG, KLHDC8A, KIR2DS4, RANBP17, LAIR2, KIR3DL1, HBQ1, GOLGA8O | KY Down MET Up 12W |
| GTEx Blood 20-29 vs 30-39 Down | 0.1048 | 6.94 | DCHS2, CELF3, KIAA1549L, RHOV, SOWAHA, FGF1, ASIC1, ENOX1 | KY Down MET Up 12W |
| GTEx Blood 20-29 vs 40-49 Up | 0.1048 | 6.94 | NLGN4Y, PIGR, TMEM178B, IFNG, ENAM, SAA4, MUC12, CCL3 | KY Down MET Up 12W |
| GTEx Blood 20-29 vs 50-59 Down | 0.4342 | 1.42 | TAS2R40, LRRN1, ACSM4, NR2E1, ASIC1 | KY Down MET Up 12W |
| GTEx Blood 20-29 vs 40-49 Down | 0.6064 | 0.65 | TAS2R40, LRRN1, AZU1, ELANE | KY Down MET Up 12W |
| GTEx Blood 20-29 vs 60-69 Down | 0.7668 | 0.23 | TAS2R40, NR2E1, ASIC1 | KY Down MET Up 12W |
| GTEx Blood 20-29 vs 70-79 Down | 0.7668 | 0.23 | TAS2R40, NR2E1, ASIC1 | KY Down MET Up 12W |
| GTEx Blood 20-29 vs 70-79 Up | 0.0003 | 42.43 | PAQR9, IL22, OR52H1, CCL4L2, ANKRD33, CCL3L3, SAA4, APOA1, BAAT, F2, BARX1, CXCL12, C9, CFHR3, CCL4, CCL3, HAO1, CTSE, SLC38A3, ACTRT3 | KY Down MET Up 24W |
| GTEx Blood 20-29 vs 60-69 Up | 0.0033 | 24.83 | ZNF683, CCL4L2, ANKRD33, KIR2DS4, SAA4, PRG2, PRG3, BAAT, ABCA13, MPO, C9, SLAMF9, CCL4, CCL3, HAO1, CTSE, ACTRT3 | KY Down MET Up 24W |
| GTEx Blood 20-29 vs 30-39 Up | 0.0069 | 20.25 | ZNF683, ALAS2, KIR2DS4, RANBP17, DEFA3, HBD, MMP8, ABCA13, NCR1, SELENBP1, ACVR1C, RNF128, OR2W3, C9, LAIR2, CTSG | KY Down MET Up 24W |
| GTEx Blood 20-29 vs 30-39 Down | 0.0306 | 12.81 | PGLYRP4, SLC45A2, SLC12A5, GOLT1A, CELF3, LRP4, SLC8A2, GAP43, CRTAC1, RHOV, SOWAHA, SH2D5, ABCG4, PDLIM4 | KY Down MET Up 24W |
| GTEx Blood 20-29 vs 40-49 Up | 0.0306 | 12.81 | CPA2, TMEM178B, CD80, CCL4L2, CCL3L3, SAA4, SLCO5A1, TSPAN12, NEU4, ENAM, CCL3, CHAC1, RNF183, SPATA18 | KY Down MET Up 24W |
| GTEx Blood 20-29 vs 50-59 Up | 0.0751 | 7.43 | LY6K, ADAMTS14, ACTN3, RNF128, C9, SAA4, CCL4, CCL3, TFF2, HAO1, BAAT, GAL3ST2 | KY Down MET Up 24W |
| GTEx Blood 20-29 vs 40-49 Down | 0.0751 | 7.43 | CA1, CEACAM6, FFAR3, DEFA3, CTSG, PRTN3, AZU1, HBD, OLFM4, MMP8, ORM2, PNPLA1 | KY Down MET Up 24W |
| GTEx Blood 20-29 vs 50-59 Down | 0.9082 | 0.08 | FFAR3, OLFM4, MMP8, TIMP4 | KY Down MET Up 24W |
| GTEx Blood 20-29 vs 60-69 Down | 0.9656 | 0.02 | PGLYRP4, SLC45A2, FFAR3 | KY Down MET Up 24W |
| GTEx Blood 20-29 vs 70-79 Down | 0.9656 | 0.02 | SLC45A2, FFAR3, GLIS2 | KY Down MET Up 24W |
| GTEx Blood 20-29 vs 30-39 Down | 0.0165 | 14.27 | CHST6, NLGN4X, LSAMP, VASH2, ADAM21, IQCD, MCHR1, WFDC2, AICDA, NFATC4, ROBO1, GJC1, CYP21A2, LGSN, TEX13C, STAC2, MAB21L2, SMCO2, ZSCAN23, MSX1, TAS2R4 | KY Up MET Down 12W |
| GTEx Blood 20-29 vs 50-59 Up | 0.0979 | 6.19 | FGB, LY6K, SPINK1, DPYS, OR51D1, CADPS, DNAJC22, GPX8, AARD, TMPRSS11E, KNG1, CYP2C9, CPS1, PAX9, CNTN4, DUOX2, SULT2A1 | KY Up MET Down 12W |
| GTEx Blood 20-29 vs 40-49 Up | 0.0979 | 6.19 | GPX8, LGALS14, TRPV3, ALDH1L2, FAM149A, WFDC2, BCL2L10, FAM161A, CPS1, CNKSR3, PAX9, IGF2BP1, SDC1, KCP, DSG2, KCNK1, CFTR | KY Up MET Down 12W |
| GTEx Blood 20-29 vs 60-69 Down | 0.2958 | 2.71 | ZNF396, OLAH, VASH2, BCDIN3D, ANO5, AICDA, ROBO1, ADAMTS2, KIAA1614, ADAMTS3, KCNMA1, TM4SF20, ECHDC3, TAS2R4 | KY Up MET Down 12W |
| GTEx Blood 20-29 vs 60-69 Up | 0.5858 | 0.88 | FGB, CYP2C9, GJB1, DAO, DPYS, AKR1D1, CPN2, KNG1, SULT2A1, SYTL2, IDO1 | KY Up MET Down 12W |
| GTEx Blood 20-29 vs 70-79 Down | 0.5858 | 0.88 | ZNF396, OLAH, ADAMTS2, KIAA1614, VASH2, BCDIN3D, L1TD1, GLI1, SCRG1, AICDA, TAS2R4 | KY Up MET Down 12W |
| GTEx Blood 20-29 vs 30-39 Up | 0.8062 | 0.32 | NCR1, FGB, CEP112, SPINK1, DPYS, REG1A, CFTR, FCAR, HOXB5 | KY Up MET Down 12W |
| GTEx Blood 20-29 vs 70-79 Up | 0.8724 | 0.17 | FGB, CYP2C9, DAO, UGT2B15, RGN, CPN2, KNG1, SULT2A1 | KY Up MET Down 12W |
| GTEx Blood 20-29 vs 50-59 Down | 0.9626 | 0.03 | OLAH, ADAMTS3, LGSN, KCNMA1, TM4SF20, VSTM1 | KY Up MET Down 12W |
| GTEx Blood 20-29 vs 40-49 Down | 0.9843 | 0.01 | OLAH, ADAMTS2, CASP5, VSTM1, FCAR | KY Up MET Down 12W |
| GTEx Blood 20-29 vs 40-49 Up | 0.0035 | 18.65 | FBXO16, GPX8, TRPV3, ALDH1L2, ETV4, WFDC2, FAM161A, CPS1, PEG10, KIF26B, RASSF6, HMSD, CNKSR3, IGF2BP1, SDC1, CLNK, DSG2, KCNN3, CCL18, STEAP1, PRSS2, CFTR, DGKI | KY Up MET Down 24W |
| GTEx Blood 20-29 vs 30-39 Down | 0.0105 | 13.19 | GABRB2, TRIM72, CHST6, NLGN4X, ZNF492, SHOX2, VASH2, PRKAG3, WFDC2, AICDA, NFATC4, ROBO1, GJC1, CSRP2, AGBL1, CYP21A2, HMSD, STAC2, ZSCAN23, HCRT, HLA-DQB2 | KY Up MET Down 24W |
| GTEx Blood 20-29 vs 70-79 Down | 0.1071 | 4.38 | CHRNB2, ZNF396, OLAH, VASH2, PARD6G, KLF14, AICDA, TCL1A, ADAMTS2, TRPV6, ZNF606, ANOS1, TNFRSF17, L1TD1, SCRG1, RAPGEF3 | KY Up MET Down 24W |
| GTEx Blood 20-29 vs 60-69 Down | 0.1700 | 3.32 | ZNF396, OLAH, CCDC121, SHOX2, VASH2, KLF14, AP3B2, AICDA, ROBO1, ADAMTS2, ANOS1, TM4SF20, TNFRSF17, AMPH, CLRN1 | KY Up MET Down 24W |
| GTEx Blood 20-29 vs 70-79 Up | 0.1700 | 3.32 | SERPINA11, TPSD1, GLYATL1, MOGAT2, UGT2B15, APOC4, KIR3DL2, ADH4, ALB, SAA1, CCL18, CCL16, HSPA1B, KIR3DX1, SULT2A1 | KY Up MET Down 24W |
| GTEx Blood 20-29 vs 50-59 Up | 0.2473 | 2.44 | TPSD1, PCDHGA2, OR51M1, DNAJC22, GPX8, FZD10, KIR3DL2, CPS1, ALB, SAA1, DUOX2, CCL16, KIR3DX1, SULT2A1 | KY Up MET Down 24W |
| GTEx Blood 20-29 vs 60-69 Up | 0.3423 | 1.74 | TPSD1, GLYATL1, AKR1D1, PON1, KIR3DL2, ADH4, ALB, CCL18, CCL16, HSPA1B, KIR3DX1, SULT2A1, IDO1 | KY Up MET Down 24W |
| GTEx Blood 20-29 vs 50-59 Down | 0.6978 | 0.48 | TSPAN16, OLAH, TRPV6, SHOX2, ANOS1, TM4SF20, VSTM1, KLF14, AP3B2, CYP17A1 | KY Up MET Down 24W |
| GTEx Blood 20-29 vs 30-39 Up | 0.8588 | 0.14 | CX3CR1, CEP112, KIR3DL2, GPRC5D, CFTR, KIR3DX1, PVALB, HOXB5 | KY Up MET Down 24W |
| GTEx Blood 20-29 vs 40-49 Down | 0.9846 | 0.01 | OLAH, ADAMTS2, VSTM1, AMPH, KLF14 | KY Up MET Down 24W |
